# Reactive Stroma as a Transversal Prognostic Biomarker for Metastasis in Breast Cancer: Integration of Digital Histopathology and Transcriptomic Profiling

**DOI:** 10.3390/ijms27052213

**Published:** 2026-02-26

**Authors:** Daniela P. Barrera, Muriel A. Núñez, Valentina Cerda I., J. Sebastián Contreras-Riquelme, Jenny Henríquez, Guillermo Carrasco, Alejandra Pereira, Vania Figueroa, Verónica Toledo, Badir Chahuan, Jorge Sapunar-Zenteno, Ximena Rodríguez, Daniel Moreno, José Tomás Larach, Benjamín Prieto, Patricia García, Leonor Moyano, José Peña, Javier Cerda-Infante

**Affiliations:** 1 Environ, Startup and Spin-Off from Pontificia Universidad Católica de Chile, Santiago 7500571, Chile; daniela@environ.bio (D.P.B.); muriel@environ.bio (M.A.N.);; 2 Facultad de Medicina y Ciencia, Universidad San Sebastián, Santiago 7510157, Chile; 3 Unidad Cirugía Oncológica Mamaria, OECI Cancer Center, Fundación Arturo López Pérez, Santiago 7500921, Chile; 4 Centro de Investigación e Innovación en Cáncer, OECI Cancer Center, Fundación Arturo López Pérez, Santiago 7500921, Chile; 5 Centro de Excelencia CIGES, Facultad de Medicina, Universidad de la Frontera, Temuco 4811230, Chile; 6 Hospital San José, Santiago 8380419, Chile; 7 Facultad de Medicina, Pontificia Universidad Católica de Chile, Santiago 8331150, Chile; 8 Red Salud UC-Christus, Santiago 8330024, Chile; 9 Department of Pathology, School of Medicine, Center for Cancer Prevention and Control (CECAN), Pontificia Universidad Católica de Chile, Santiago 8330077, Chile; 10 Instituto Nacional del Cáncer, Santiago 8380450, Chile

**Keywords:** reactive stroma, tumor microenvironment, metastasis, digital pathology, cancer prognosis

## Abstract

Distant metastasis is the main cause of breast cancer (BC) mortality, yet current prognostic models remain largely tumor-centric and underutilize stromal biology. In this study, we quantified reactive stroma, a collagen-rich and fibrotic fraction of the stromal compartment, as a subtype-independent biomarker of metastatic risk. A retrospective cohort of 182 FFPE primary BC biopsies (2006–2020) was analyzed. Total stroma was quantified on H&E-stained sections and reactive stroma on Masson’s trichrome using QuPath with pathologist validation. Cutoffs were defined using maximally selected rank statistics, and overall survival (OS) and metastasis-free survival (MFS) were evaluated by Kaplan–Meier analysis and multivariable Cox regression. RNA sequencing was performed in a subset of cases to characterize associated transcriptomic programs. While total stromal content showed univariate associations with OS and MFS, it was not independently prognostic after adjustment. In contrast, high reactive stroma (cutoff 53.2%) independently predicted shorter MFS (HR = 3.76; *p* < 0.001), irrespective of molecular subtype and clinicopathological variables. Tumors with high reactive stroma exhibited upregulation of extracellular matrix and profibrotic genes (including FN1, OLR1, and EDN2), enrichment of collagen remodeling and TGF-β signaling pathways, and reduced T-cell activation signatures. These findings demonstrate that quantitative assessment of reactive stroma from standard histological stains is a reproducible, subtype-independent biomarker of metastatic risk in BC and can be readily integrated into routine pathology workflows to improve risk stratification.

## 1. Introduction

Breast cancer (BC) remains one of the leading causes of cancer-related mortality worldwide, with distant metastasis accounting for most deaths [1]. In 2022, GLOBOCAN reported over 2.3 million new cases of BC and more than 685,000 related deaths globally. Despite advances in diagnosis and treatment, both incidence and mortality continue to rise, largely due to metastatic progression, even among patients treated with curative intent [2]. This highlights the urgent need for new prognostic biomarkers capable of accurately predicting metastatic risk at the time of diagnosis.

Currently available prognostic biomarkers in BC are primarily based on histopathological and molecular features, such as hormone receptor status, HER2 expression, tumor size, and lymph node involvement [3,4]. While these markers guide therapeutic decisions, they have significant limitations in predicting the actual risk of metastasic dissemination. Even additional indicators, such as the Ki-67 proliferation index or commercial multigene assays, often fail to accurately forecast metastatic progression across all subtypes [5,6]. This shortcoming represents a critical gap in the early clinical management of patients, where more precise risk stratification could directly inform therapeutic decision-making.

The tumor microenvironment (TME), particularly its stromal compartment, has emerged as a key driver of tumor progression [7,8]. This compartment includes cancer-associated fibroblasts (CAF), immune cells, and extracellular matrix (ECM) proteins, which collectively interact dynamically with tumor cells [9,10,11,12,13,14,15,16]. CAF play a central role in TME remodeling by secreting ECM proteins, growth factors, and cytokines, thereby promoting tumor invasion, angiogenesis and immune evasion [14,17,18,19]. These functions contribute to the transformation of the stroma into a reactive state, characterized by a fibrotic and proinflammatory phenotype that co-evolves with the tumor and facilitates metastasis progression.

Reactive stroma is histologically defined as a fibrotic and proinflammatory microenvironment that emerges during early stages of carcinogenesis. It is characterized by the activation of fibroblasts into a myofibroblast-like phenotype, with increased synthesis of ECM components such as type I collagen, tenascin-C and fibronectin, as well as the expression of mesenchymal markers like vimentin and fibroblast activation protein alpha (FAP-α) [20,21]. This response, resembling chronic wound repair, promotes tissue stiffness, stromal architectural remodeling, and tumor progression. Despite its recognized biological significance, the specific quantification of reactive stroma and its prognostic value in breast cancer remain insufficiently characterized—partly due to the difficulty of distinguishing it from total stroma in conventional assessments.

Although the tumor–stroma ratio (TSR) has been widely validated as a poor prognostic factor in breast cancer, it conceptualizes the stroma as a structurally homogeneous compartment without accounting for its functional heterogeneity [22,23,24]. This is particularly important considering that reactive stroma represents a biologically active fraction of the tumor microenvironment that not only responds to tumor signals but co-evolves with it, promoting proliferation, immune evasion, invasion, and metastatic spread [25,26,27]. Histologically, reactive stroma is characterized by increased collagen deposition, fibroblast hypercellularity, and extensive extracellular matrix remodeling [26].

However, its clinical quantification has been limited by the lack of objective and standardized methodologies capable of distinguishing it from the remaining stromal content. While some studies have employed immunohistochemical markers such as FAP-α or α-SMA to detect activated [26,28], these approaches require additional validation and rely on specialized platforms not readily available in routine clinical settings. In contrast, our methodological strategy proposes a feasible and reproducible approach using conventional stains: hematoxylin–eosin (H&E) to quantify total stromal content and Masson’s trichrome to identify collagen-rich regions indicative of reactive stroma, analyzed via digital pathology tools. This allows the calculation of the reactive stroma percentage relative to total stroma in each tumor sample, yielding an objective metric with potential clinical prognostic value.

To address this gap, we implemented a differential approach based on the automated and validated quantification of reactive stroma as a proportion of the total stromal area in each tumor sample. We used standard histological stains, H&E for total stroma and Masson’s trichrome for collagen-rich reactive stroma, on primary formalin-fixed, paraffin-embedded (FFPE) tumor biopsies from patients with breast cancer spanning all major molecular subtypes and analyzed the slides using QuPath digital pathology software. This methodology enables the objective and reproducible distinction between total stromal content and its fibrotic, biologically active fraction.

The main objective of this study was to determine whether histologically defined reactive stroma can serve as a metastasis-specific prognostic biomarker in breast cancer, independently of molecular subtype. We evaluated its association with overall survival (OS) and metastasis-free survival (MFS) and investigated its molecular underpinnings through transcriptomic profiling. By integrating digital pathology with transcriptomic analysis of routinely available clinical samples, this study proposes a scalable and clinically relevant strategy for early risk stratification and the potential development of stromal-targeted therapeutic approaches.

## 2. Results

### 2.1. Clinical and Clinicopathological Characteristics of the Breast Cancer Cohort

To characterize the patient cohort prior to molecular and clinical analyses, 182 patients with invasive breast cancer were included, with a median follow-up of 60 months. All patients met the following inclusion criteria: available FFPE tumor samples, available clinical and follow-up information for survival analyses, and no history of neoadjuvant therapy prior to biopsy. Patients with a previous diagnosis of cancer or hereditary cancer syndromes were excluded from the study.

H&E-stained sections were obtained for all 182 patients, with overall survival (OS) data available for all patients and metastasis-free survival (MFS) data available for 134 patients. A total of 146 cases were analyzed via Masson’s trichrome staining, with complete OS and MFS data available for 85 patients. Clinicopathological characteristics, including age, tumor size (pT), nodal status (pN), molecular subtypes (ER, PR, HER2), and histological grade, are summarized in Table 1.

### 2.2. Quantification of Total Stromal Content in Breast Cancer Tissue

**Figure 1:**
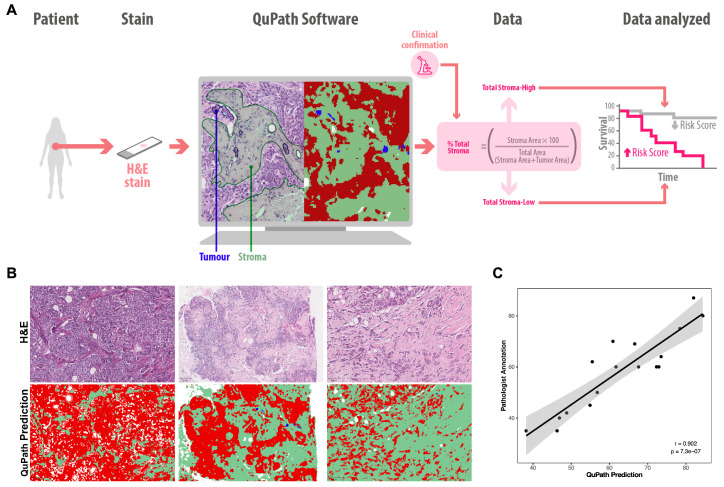
Quantification of the total stroma percentage in BC. (**A**) Flowchart illustrating the methodology used to quantify the total stroma percentage in FFPE breast tumor tissue samples. The samples were stained with H&E and analyzed via QuPath software to identify the stroma and tumor areas, with validation performed by a pathologist. (**B**) Representative images of H&E staining (top) and corresponding QuPath predictions (bottom) in samples with low, medium, and high total stroma percentages. (**C**) Correlation between total stroma quantification by QuPath and pathologist validation (*n* = 17). Each dot represents an individual sample; the solid line indicates the linear regression, and the gray shaded area represents the 95% confidence interval.

To evaluate whether the amount of total stroma present in tumor tissue was associated with clinical outcomes, the percentage of stromal area was quantified in FFPE sections stained with H&E via QuPath image analysis software (v0.5.0). A supervised learning approach was applied in which manual annotations were used to train a pixel classifier capable of discriminating between stromal, tumor, and non-stromal regions. This enabled automated segmentation and precise quantification of each tissue compartment. QuPath uses pixel-level classification on the basis of specific visual characteristics defined by initial annotations. This process allowed the identification and delineation of relevant tissue components, including the stromal area (green) and tumor or other structures (red), in each digital sample (Figure 1A). The total stromal content was calculated as the ratio of the stromal area to the total tissue area (Figure 1A,B). The automated method was validated against manual segmentation performed by two independent pathologists in a subset of 17 cases, which showed high concordance (r = 0.902; *p* < 0.001) (Figure 1C).

### 2.3. Association Between Total Stromal Content and Patient Survival

**Figure 2:**
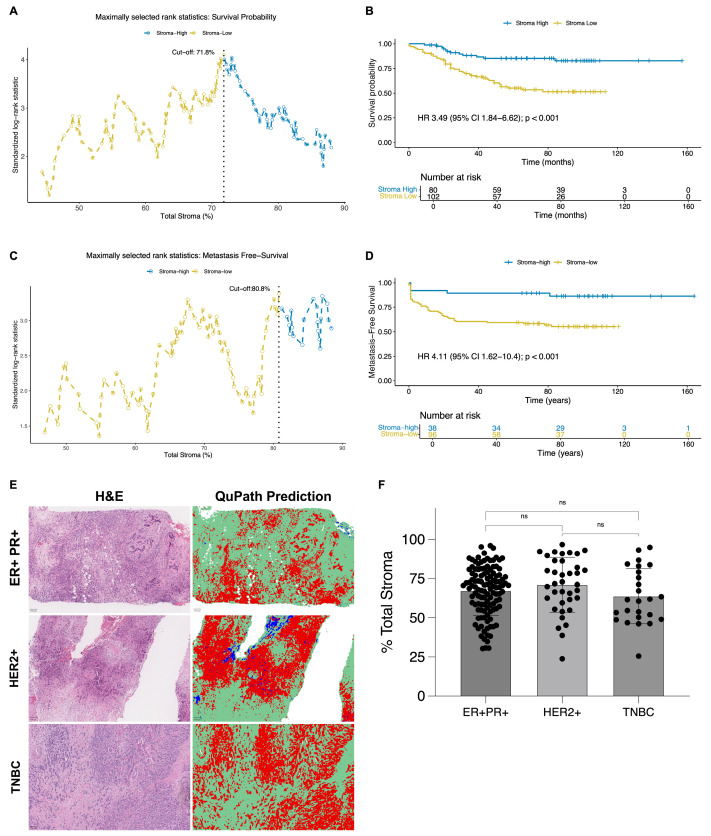
Associations of total stromal content with clinical outcomes and molecular subtypes in patients with breast cancer. (**A**,**B**) Maximally selected rank statistics and Kaplan–Meier survival analysis for overall survival (OS) in patients with breast cancer stratified by total stromal content (*n* = 182). (**A**) Determination of the optimal cutoff value (71.8%) via maximally selected rank statistics. (**B**) Kaplan–Meier curve showing significantly shorter OS in patients with low stromal content (HR 3.49; 95% CI: 1.84–6.62; *p* < 0.001). (**C**,**D**) Analysis of metastasis-free survival (MFS) in a subset of patients (*n* = 134). (**C**) The optimal cutoff value for MFS was 80.8%. Dashed vertical lines in panels (**A**,**C**) indicate the respective cutoff values. (**D**) Kaplan–Meier curve indicating significantly worse MFS in the Stroma-Low group (HR 4.11; 95% CI: 1.62–10.4; *p* < 0.001). (**E**) Representative H&E-stained images and corresponding QuPath-based segmentation of stromal and tumor areas in tumors classified as ER+/PR+, HER2+, or triple-negative breast cancer (TNBC). Stromal areas are shown in green, tumor (non-stromal) regions in red, and other cellular components in blue. (**F**) Percentage of total stromal content across molecular subtypes (ER+/PR+, HER2+, and TNBC) quantified from H&E-stained sections via QuPath. No statistically significant differences were observed among the subtypes (ns, not significant; Kruskal–Wallis test).

Maximally selected rank statistical analysis identified an optimal cutoff of 71.8% total stromal content for overall survival (OS) stratification (Figure 2A). In the study cohort, the total stromal content ranged from 23.9% to 96.7%, with a mean of 67.8% and a standard deviation of 16.5%. On the basis of this threshold, 102 patients were classified as having low total stroma, and 80 patients were classified as having high total stroma, corresponding to 56.0% and 44.0% of the cohort, respectively.

Patients in the low total stroma group (≤71.8%) exhibited significantly shorter OS in the univariate analysis (HR = 3.49; 95% CI: 1.84–6.62; *p* < 0.001) (Figure 2B; Supplementary Table S1). However, this association was no longer statistically significant after adjusting for age, tumor size (pT), nodal status (pN), molecular subtype, or histological grade (HR = 2.49; 95% CI: 0.60–10.35; *p* = 0.209) (Supplementary Table S2), indicating that its prognostic effect may be influenced by other clinicopathological variables. Given that distant metastasis is the leading cause of breast cancer-related mortality, we examined whether the total stromal content was associated with metastasis-free survival (MFS).

For MFS, the maximally selected rank statistical analysis identified an optimal cutoff of 80.8% total stroma content (Figure 2C). In this subset, the total stromal content ranged from 23.9% to 96.7%, with a mean of 69.9% and a standard deviation of 16.3%. On the basis of this threshold, 96 patients were classified as having low total stroma (≤80.8%), and 38 were classified as having high total stroma (>80.8%), accounting for 71.6% and 28.4% of the MFS cohort, respectively.

Patients in the low total stroma group exhibited a significantly shorter MFS in the univariate analysis (HR = 4.11; 95% CI: 1.62–10.40; *p* < 0.001) (Figure 2D, Supplementary Table S1), and this trend persisted in the multivariate model, although it did not reach statistical significance (HR = 4.08; 95% CI: 0.88–18.94; *p* = 0.073) (Supplementary Table S2).

Although the association between low total stromal content and poor prognosis was significant in the univariate analyses for both OS and MFS, it did not remain statistically significant in the multivariate models after adjusting for age, tumor size, nodal status, molecular subtype, and histological grade. To determine whether the prognostic relevance of total stromal content might be influenced by molecular subtype, we analyzed its distribution across ER+/PR+, HER2+, and triple-negative tumors. Quantitative analysis of H&E-stained images via QuPath revealed no statistically significant differences in stromal content among subtypes (Figure 2E,F), indicating that total stromal content is not associated with molecular subtype and constitutes a consistent feature of the tumor microenvironment across different biological contexts.

### 2.4. Transcriptional Features Associated with Total Stromal Content

Given the lack of significant associations in multivariate models and the observation that total stromal content does not vary across molecular subtypes, we investigated the biological nature of the stromal compartment itself. While total stroma provides a quantitative measure of tissue microarchitecture, it may not fully capture the functional activity of stromal cells involved in tumor progression. To address this, we evaluated the expression of genes commonly expressed by CAF and other stromal components. Specifically, we assessed the expression of VIM, PDGFRA, PDGFRB, and ACTA2, which are genes predominantly expressed by stromal cells, particularly CAF, and are involved in mesenchymal activation and extracellular matrix remodeling, via RNA sequencing of a representative subset of the same FFPE samples used for stromal quantification.

**Figure 3:**
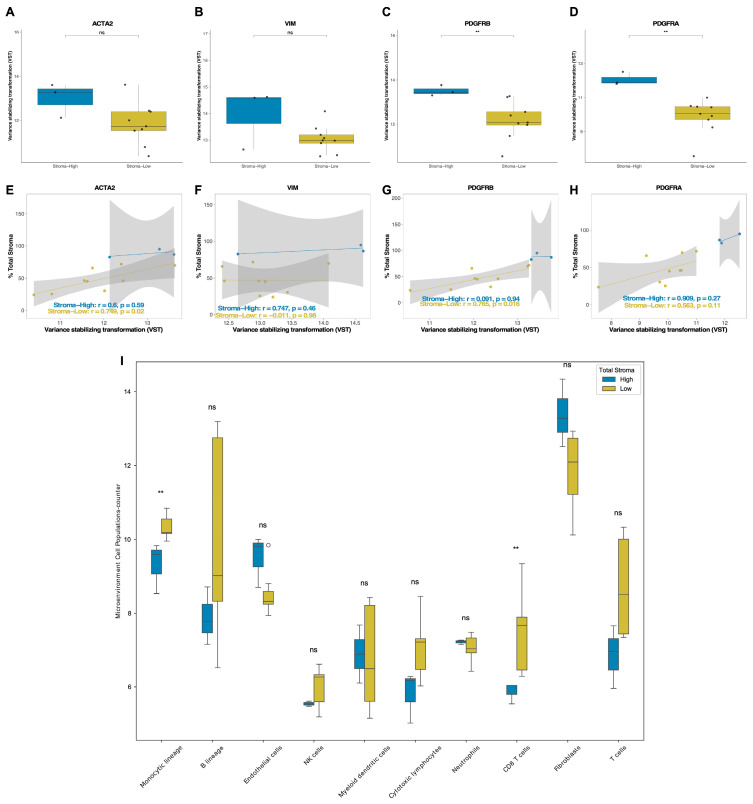
Expression of CAF-associated genes and the abundance of tumor microenvironment cell populations according to the total stromal content in breast cancer. (**A**–**D**) Box plots showing the variance-stabilized expression levels of the CAF markers VIM (**A**), PDGFRA (**B**), PDGFRB (**C**), and ACTA2 (**D**) in tumors classified as Stroma-High and Stroma-Low based on the total stromal percentage. (**E**–**H**) Scatter plots illustrating the correlation between VST-normalized gene expression and total stromal area across samples. Pearson’s r and *p*-values are shown for each stromal group. (**I**) MCP-counter analysis showing the estimated abundance of tumor microenvironment cell populations in patients with high and low total stromal content. (ns, not significant; ** *p* < 0.01; Wilcoxon rank-sum test).

Among these genes, PDGFRA and PDGFRB were significantly upregulated in tumors classified as total stroma-high (*p* < 0.05) (Figure 3A–D). Although VIM and ACTA2 were more highly expressed in the total stroma-high group, these differences were not statistically significant. Moreover, correlation analysis revealed that gene expression levels were strongly positively correlated with the stromal percentage in the total stroma-low group (Figure 3E–H), suggesting a complex relationship between stromal abundance and transcriptional activity. In addition, MCP-counter analysis revealed higher fibroblast scores in tumors with high total stromal content (Figure 3I), reflecting increased infiltration of stromal cell populations in these samples.

Although low total stromal content was significantly associated with poorer OS and MFS, these results indicate that the total stromal percentage does not necessarily reflect the abundance or activation state of CAF. Given that CAF play a pivotal role in tumor progression, we hypothesized that the total stromal content may fail to capture the functionally active stromal compartment. Therefore, we quantified the reactive stroma, defined as the fraction of stroma characterized by a densely remodeled ECM enriched in activated CAF with a myofibroblastic phenotype and high secretory activity of protumorigenic factors, including collagen types I and II. This component represents the biologically active portion of the tumor stroma and is directly involved in invasion, angiogenesis, immunosuppression, and therapeutic resistance. Its quantification enabled a more specific assessment of its prognostic relevance. Together, these results indicate that total stromal content alone does not adequately reflect the functional activation state of CAF, prompting the need for a more biologically specific stromal metric.

### 2.5. Quantification of Reactive Stroma as a Functional Stromal Biomarker

**Figure 4:**
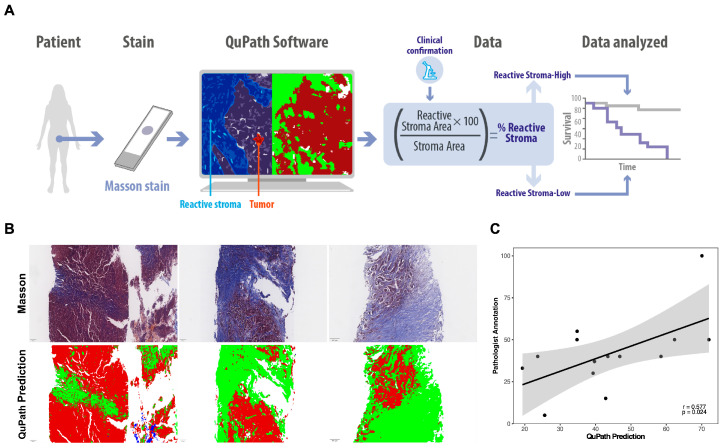
Quantification of reactive stroma in BC. (**A**) Flowchart illustrating the methodology used to quantify the percentage of reactive stroma relative to total stroma in FFPE breast tumor tissue samples. The samples were stained with Masson’s trichrome and analyzed via QuPath software to identify the tumor and reactive stroma areas, followed by clinical validation by a pathologist. (**B**) Representative images of Masson’s trichrome staining (top) and corresponding QuPath predictions (bottom) in samples with low, medium, and high percentages of reactive stroma. (**C**) Correlation between reactive stroma quantification by QuPath and validation by pathologists (*n* = 14).

To capture the biologically active stromal compartment associated with tumor progression, reactive stroma was quantified in 146 FFPE breast cancer samples stained with Masson’s trichrome by measuring the collagen-rich extracellular matrix area relative to the total stromal area previously identified in H&E-stained sections. This quantification was performed using QuPath software with supervised pixel classification, enabling discrimination of reactive stroma, tumor, and other tissue structures (Figure 4A,B). The automated approach was validated in a subset of 14 cases by comparison with manual annotations performed by expert pathologists, revealing a significant correlation between methods (r = 0.577; *p* < 0.05) (Figure 4C).

### 2.6. Reactive Stroma Stratification and Its Association with Patient Survival

**Figure 5:**
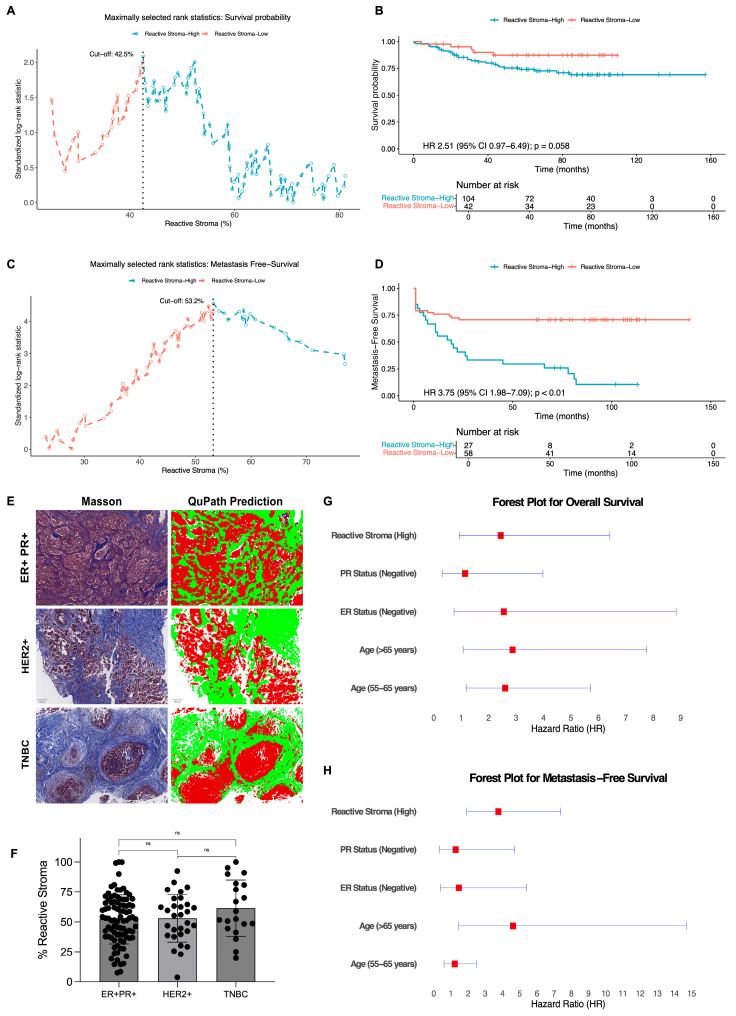
Associations of the reactive stromal content with clinical outcomes, molecular subtypes, and multivariate prognostic value in breast cancer. (**A**,**B**) Maximally selected rank statistics and Kaplan–Meier analysis for overall survival (OS) based on the percentage of reactive stroma (*n* = 146). (**A**) Optimal cutoff point identified at 42.5% reactive stroma. (**B**) Kaplan–Meier curve showing a trend toward worse OS in patients with highly reactive stroma (HR = 2.51; 95% CI: 0.97–6.49; *p* = 0.058). (**C**,**D**) Analysis of metastasis-free survival (MFS) in a subset of patients (*n* = 85). (**C**) Optimal cutoff point determined at 53.2% reactive stroma. Dashed vertical lines in panels (**A**,**C**) indicate the respective cutoff values. (**D**) Kaplan–Meier curve demonstrating a significantly lower MFS in the reactive stroma-high group (HR = 3.75; 95% CI: 1.98–7.09; *p* < 0.01). (**E**) Representative Masson’s trichrome-stained images and corresponding QuPath segmentation of reactive stroma in ER+/PR+, HER2+, and triple-negative breast cancer (TNBC) tumors. (**F**) Quantitative comparison of the reactive stromal content across molecular subtypes revealed no significant differences, suggesting that the reactive stroma is a subtype-independent feature. (ns, not significant; Kruskal–Wallis test). (**G**,**H**) Forest plots of multivariate Cox regression analyses for OS (**G**) and MFS (**H**). In forest plots, red squares represent hazard ratios (HRs) and horizontal lines indicate 95% confidence intervals.

To determine whether the biologically active stromal compartment has prognostic relevance beyond total stromal abundance, patients were stratified according to the percentage of reactive stroma quantified in Masson’s trichrome–stained sections. Maximally selected rank statistical analysis identified optimal cutoffs of 42.5% and 53.2% reactive stromal content for OS and MFS, respectively (Figure 5A,C).

In the OS cohort (*n* = 146), the percentage of reactive stromal content ranged from 3.7% to 100%, with a mean of 55.2% and a standard deviation of 21.9%. Based on the 42.5% cutoff, 42 patients were classified as reactive stroma–low, and 104 patients as reactive stroma–high. For the MFS analysis, a subset of 85 patients from the same cohort with available time to metastatic progression was included. In this subgroup, the reactive stromal content ranged from 14.5% to 100%, with a mean of 48.7% and a standard deviation of 21.0%. According to the 53.2% threshold, 58 patients were assigned to the Reactive Stroma–Low group, and 27 were assigned to the Reactive Stroma–High group. This stratification enabled a more specific evaluation of the prognostic impact of the biologically active stromal compartment, providing insights beyond those obtained from total stromal quantification.

According to the univariate analysis, patients in the Reactive Stroma–High group tended to have shorter OS (HR = 2.51; 95% CI: 0.97–6.49; *p* = 0.058) (Figure 5B, Supplementary Table S3) and significantly shorter MFS (HR = 3.75; 95% CI: 1.98–7.09; *p* < 0.01) (Figure 5D, Supplementary Table S3). These findings support the hypothesis that CAF-enriched reactive stroma, rather than stromal quantity alone, represents a biologically relevant driver of tumor progression.

We also evaluated whether the reactive stromal content varied among different molecular subtypes of breast cancer. No statistically significant differences were observed among the ER+/PR+, HER2+, and triple-negative breast cancer (TNBC) groups (Figure 5E,F), supporting the notion that reactive stroma constitutes a consistent and subtype-independent feature of the breast cancer tumor microenvironment.

Importantly, multivariate Cox regression analysis confirmed that high reactive stromal content remained an independent prognostic factor for MFS after adjusting for age, ER status, and PR status (HR = 3.76; 95% CI: 1.91–7.39; *p* < 0.001), whereas its association with OS showed a non-significant trend (HR = 2.44; 95% CI: 0.93–6.4; *p* = 0.069) (Figure 5G,H, Table 2). Together, these results provide robust evidence that reactive stroma captures biologically and clinically meaningful features of the tumor microenvironment that are not reflected by conventional molecular markers. The strong and independent association with metastatic risk highlights its potential utility as a prognostic biomarker in breast cancer and supports the incorporation of stromal biology into future risk stratification strategies.

### 2.7. Transcriptomic Programs Associated with Reactive Stromal Content

Given the strong prognostic value of histologically defined reactive stroma, we next explored whether this phenotype is associated with distinct transcriptomic programs linked to tumor progression. To address this question, RNA sequencing (RNA-Seq) was performed on 12 FFPE tumor samples previously characterized histologically and selected based on high or low reactive stromal content. Differential gene expression (DEG) analysis was conducted between tumors with high and low reactive stroma, followed by gene set enrichment analysis (GSEA) and functional annotation using the KEGG and Reactome databases, to identify biological pathways associated with the reactive stromal phenotype.

**Figure 6:**
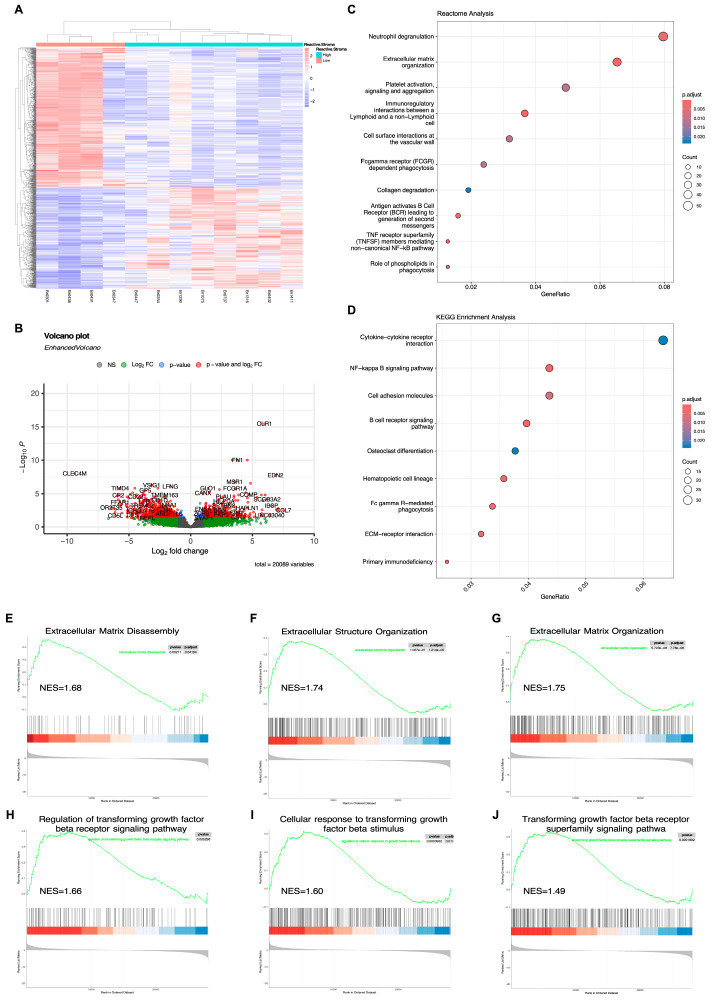
Gene expression and pathway enrichment analyses associated with reactive stromal content. (**A**) Heatmap of DEGs between tumors with high and low reactive stromal content. The samples are clustered according to the stromal phenotype. (**B**) Volcano plot from DEG analysis. Genes with statistically significant differences (adjusted *p*-value < 0.05) and | log2(fold change) | > 1 are shown in red; genes meeting only the fold-change threshold are shown in green, genes meeting only the *p*-value threshold in blue, and non-significant genes in grey. (**C**) Reactome pathway enrichment analysis revealed that tumors with high reactive stromal content were enriched in pathways related to Fc gamma receptor-mediated phagocytosis, collagen degradation, platelet activation, and extracellular matrix organization. (**D**) KEGG pathway enrichment analysis showing immune-related and extracellular matrix-associated pathways enriched in tumors with high reactive stromal content. (**E**–**G**) Gene set enrichment analysis (GSEA) plots for the terms extracellular matrix disassembly (**E**), extracellular matrix organization (**F**), and extracellular structure organization (**G**), indicating significant upregulation of these processes in tumors with highly reactive stroma. (**H**–**J**) GSEA plots for the terms regulation of the transforming growth factor beta receptor signaling pathway (**H**), cellular response to transforming growth factor beta stimulus (**I**), and the transforming growth factor beta receptor superfamily signaling pathway (**J**), suggesting transcriptional upregulation of TGF-β–associated programs in tumors with high reactive stromal content.

To visualize global transcriptomic differences, a heatmap generated using representative differentially expressed genes revealed a clear separation between tumors with high and low reactive stromal content (Figure 6A), indicating distinct transcriptional states associated with this stromal phenotype. Consistently, the volcano plot highlighted multiple differentially expressed genes showing large effect sizes and statistical significance (adjusted *p*-value < 0.05; |log2-fold change| > 1) (Figure 6B). Among the genes upregulated in tumors with high reactive stromal content were FN1, OLR1, EDN2, and MSR1, which are implicated in extracellular matrix remodeling, inflammatory signaling, and modulation of the tumor microenvironment—processes closely linked to invasion and metastatic dissemination.

Pathway enrichment analysis using the Reactome database revealed significant overrepresentation of ECM-related and immune-associated pathways, including collagen degradation, ECM activation, and ECM organization, in tumors with high reactive stromal content (Figure 6C). These findings were independently supported by KEGG analysis, which identified enrichment of pathways such as ECM–receptor interaction, cell adhesion molecules, NF-κB signaling, and B-cell receptor signaling in the reactive stroma–high group (Figure 6D).

To further validate these results at the gene set level, GSEA focusing on ECM-related biological processes demonstrated significant positive enrichment of extracellular matrix disassembly (NES = 1.68), extracellular structure organization (NES = 1.74), and extracellular matrix organization (NES = 1.75) in tumors with high reactive stromal content (Figure 6E–G). In addition, multiple gene sets related to TGF-β signaling, including regulation of the TGF-β signaling pathway (NES = 1.66), cellular response to TGF-β stimulus (NES = 1.60), and transforming growth factor beta receptor superfamily signaling (NES = 1.49), were positively enriched (Figure 6H–J), consistent with activation of profibrotic and tissue-remodeling programs.

Conversely, significant negative enrichment of immune-related gene sets, including T-cell receptor signaling, positive regulation of T-cell activation, leukocyte cell–cell adhesion, and T-cell proliferation, was observed in tumors with high reactive stromal content (Supplementary Figure S1), suggesting suppression of adaptive immune responses within a highly reactive stromal microenvironment. Together, these transcriptomic findings provide a mechanistic framework linking reactive stroma to extracellular matrix remodeling, immune modulation, and the adverse clinical outcomes observed in patients with high reactive stromal content.

## 3. Discussion

### 3.1. Prognostic Value of Reactive Stroma in Breast Cancer

The TME has emerged as a key driver of cancer progression [29,30]. In this context, our study demonstrated that the proportion of reactive stroma represents an independent prognostic variable in patients with breast cancer. Tumors enriched in reactive stroma exhibited significantly shorter metastasis-free survival (MFS) than those enriched with lower stromal content, a finding that remained robust after adjustment for age and hormone receptor status. These results provide prognostic information beyond current clinicopathological markers, highlighting that reactive stroma assessment can refine risk stratification independently of traditional factors.

Importantly, our measurements are reproducible and objective, enabling consistent application across institutions and patient cohorts. This reinforces the potential for multicenter standardization and broad clinical adoption.

### 3.2. Histological Characterization and Methodological Strengths

Reactive stroma, originally described in prostate cancer [20], is characterized by the activation of fibroblasts and the deposition of ECM proteins such as type I collagen, tenascin, and fibronectin. Abundant ECM accumulation not only provides structural support but also actively promotes tumor progression. Excessive ECM accumulation has been associated with poor prognosis across multiple solid tumors [31,32,33], emphasizing the functional relevance of stromal remodeling in cancer biology. However, desmoplasia, or reactive stroma, is often acellular or composed predominantly of the extracellular matrix [34,35]. This characteristic may limit the detection of specific cellular transcripts in bulk RNA-Seq analyses. Therefore, transcriptional profiles derived from tumors with extensive acellular stroma should be interpreted cautiously, particularly when assessing cell-specific gene expression.

In our study, samples were quantified via Masson’s trichrome staining, combined with digital image analysis for precise, standardized, and objective segmentation of collagen-rich ECM fractions. This approach enables reproducibility across laboratories and institutions, ensuring that measurements can be directly compared and validated in different clinical settings. Such methodological robustness strengthens the validity of linking stromal content to clinical outcomes and molecular alterations, facilitating its integration into routine pathology workflows without major procedural changes.

### 3.3. Biological Mechanisms Linking ECM Remodeling to Metastasis

Several studies have shown that collagen-enriched ECM promotes tumor progression and metastatic dissemination [36,37]. Although previous studies have suggested that luminal tumors (ER+/HER2−) present greater stromal content than triple-negative breast cancer (TNBC) [38], our analysis did not reveal significant differences in total or reactive stroma among molecular subtypes. This finding indicates that reactive stroma is a subtype-independent across breast cancer subtypes, reinforcing its prognostic significance beyond molecular classification and current clinicopathological markers.

A detailed examination of the reactive stromal compartment revealed significant associations with MFS, supporting the view that reactive stroma is not a subtype-specific phenomenon but a functionally active component of the TME broadly implicated in metastatic risk. The biological basis for this prognostic role is grounded in the functional properties of its major constituents, particularly type I collagen. Accumulated collagen promotes cell migration through the activation of integrins and receptors, such as DDR1, triggering cytoskeletal reorganization and acquisition of an invasive phenotype [39,40,41,42]. Increased collagen density induces tissue stiffness, which modulates cellular behavior through mechanotransduction pathways, most notably through focal adhesion kinase (FAK) activation [43,44]. This process triggers downstream cascades, including ERK activation, Rho-GTPase signaling, and integrin clustering, promoting cellular contractility, invasion, and morphological plasticity [43,45]. These dynamic interactions between tumor cells and the ECM contribute to tissue remodeling, altered cell–cell and cell–ECM adhesion, and ultimately drive tumor cells toward an invasive phenotype capable of penetrating the matrix and initiating the metastatic cascade [43,44,45,46]. Our findings reinforce the concept that, in addition to its architectural role, collagen content constitutes a biologically active component of the tumor stroma that is associated with poor prognosis.

### 3.4. Clinical Relevance of Stromal Biomarkers

Our findings reinforce the concept that collagen content, beyond its architectural role, is a biologically active component of the tumor stroma associated with poor prognosis. In prostate cancer, Ruder et al. developed a quantitative reactive stroma (qRS) biomarker independently associated with increased disease-specific mortality and biochemical recurrence [47]. In breast cancer, Sharma et al. validated Stratipath Breast, which has strong prognostic performance in ER+/HER2− patients (HR = 2.76; *p* < 0.001) [48]. While stratification focuses on predicting general disease progression, our study specifically addresses metastasis, demonstrating that reactive stroma ≥53.2% was associated with a significantly shorter MFS (HR = 3.75; *p* < 0.01), and reactive stroma ≥42.5% was associated with a trend toward worse OS (HR = 2.51; *p* = 0.058).

Importantly, assessing reactive stroma through Masson’s trichrome staining combined with digital image analysis offers a simple, reproducible, and objective method. This quantification is based on standardized, automated segmentation, enabling consistent application across institutions and supporting multi-center validation. Unlike complex molecular assays, reactive stroma evaluation can be readily integrated into routine diagnostic workflows with minimal procedural modification, while providing prognostic information that complements and extends beyond current markers.

### 3.5. Integration with Transcriptomic Profiling

A major strength of our study is the integration of digital pathology with transcriptomic profiling of FFPE tumor tissue. Although transcriptomic data validated the activation of fibroblast-related and ECM remodeling pathways, they remain complementary to histological quantification. RNA-Seq of a subset of histologically characterized tumors, we identified a transcriptional signature enriched in biological programs associated with fibroblast activation and ECM remodeling. Notably, tumors with high reactive stroma content exhibited significant overexpression of genes such as FN1, OLR1, EDN2, and MSR1. FN1, which encodes fibronectin, is a glycoprotein expressed in the ECM as a dimer or polymer [49,50]. This protein plays a pivotal role in physiological processes, such as wound healing, and pathological processes, such as tumor progression, facilitating cell adhesion, migration, and invasion [51,52]. In line with our histological findings, fibronectin is deposited as fibrillar complexes that reorganize the ECM, generating a dense and stiff matrix enriched in type I collagen. This structural reorganization promotes mechanotransduction, activates pro-oncogenic signaling pathways such as FAK and YAP, and enhances cell migration, immune evasion, and therapy resistance [53,54].

The correlation between FN1 overexpression and high reactive stroma content supports the concept that the ECM is not merely a static scaffold but also an active participant in tumor progression. Specifically, we demonstrated that tumors with abundant type I and III collagen synthesized by CAF presented a greater risk of metastasis, underscoring the functional importance of the stromal compartment in disease dissemination.

Consistently, we also observed the overexpression of OLR1, a receptor for oxidized lipoproteins (oxLDLs), which has previously been implicated in promoting a protumoral CAF phenotype. OLR1 activation stimulates fibroblast activation, collagen synthesis, and ECM remodeling, creating a dense and mechanically active microenvironment that facilitates tumor cell migration and immune evasion [55]. Additionally, OLR1 expression has been associated with increased infiltration of M2 macrophages and upregulation of immune checkpoint molecules such as PD-L1 [56], further reinforcing its role in establishing an immunosuppressive TME.

Similarly, the overexpression of EDN2, a member of the endothelin family and an adverse prognostic marker in breast cancer, was observed. EDN2 promotes tumor microenvironment remodeling through proliferation, migration, and activation of pro-oncogenic pathways such as the STAT3 pathway [57].

In addition to individual genes, functional pathway enrichment analyses provided broader insights into stromal activity. Reactome and KEGG analyses revealed significant enrichment of ECM-related pathways, including those related to collagen degradation, ECM organization, and ECM–receptor interactions, emphasizing the functional involvement of the stroma in poor-prognosis tumors. Notably, the TGF-β signaling pathway was also enriched in tumors with highly reactive stroma. TGF-β is a well-established driver of fibroblast-to-myofibroblast transition and ECM deposition, and its persistent activation within the TME has been linked to epithelial-mesenchymal transition (EMT), immunosuppression, and increased metastatic potential [58,59,60,61,62].

Conversely, we observed negative enrichment of immune-related gene sets, including those related to T-cell receptor signaling, T-cell proliferation, and leukocyte adhesion [63,64]. These findings suggest that CAF-mediated ECM remodeling not only promotes invasion but also restricts immune cell infiltration, fostering an immunosuppressive tumor microenvironment. Future studies could also leverage digital pathology and machine learning algorithms to incorporate additional stromal parameters such as fiber density, length, linearity, and thickness. Standardizing the measurement of these features across institutions would improve reproducibility, ensure objectivity, and allow multi-center validation. The quantitative assessment of these features could refine risk stratification and enhance the prognostic utility of the reactive stroma, providing clinically relevant information beyond existing biomarkers and enabling integration into routine diagnostic workflows.

In summary, our transcriptomic results reinforce the concept that the reactive stroma is a biologically active compartment fostering an invasive and immune-suppressive tumor phenotype. The integration of molecular findings with histological quantification provides a mechanistic rationale for its prognostic value and highlights its potential as a clinical biomarker in breast cancer.

While our study benefits from a robust cohort (*n* = 182) and a median follow-up of 60 months, future validation in independent, multi-institutional series will further strengthen its generalizability. Our use of Masson’s trichrome staining with digital segmentation offers precise, reproducible measurements of the ECM and could be complemented by multiplex immunohistochemistry or spatial transcriptomics to refine the cellular resolution within the stroma. Finally, as treatment paradigms have evolved over the 2006–2020 period, ongoing investigations incorporating contemporary therapeutic regimens will ensure continued relevance to current clinical practice.

Taken together, our findings provide a comprehensive characterization of the reactive stromal compartment in breast cancer, highlighting its dual role as a structural and signaling component that promotes metastasis. By combining histological and transcriptomic evidence, we establish the reactive stroma as an integral feature of the tumor microenvironment with both prognostic and biological significance. These insights offer a compelling rationale for incorporating stromal assessment into routine diagnostic workflows and for the development of stromal-targeted therapies. As the field moves toward more personalized oncology, evaluating stromal dynamics may refine risk stratification and guide treatment decisions beyond conventional tumor cell-centric models.

## 4. Materials and Methods

### 4.1. Study Design and Patient Characteristics

This retrospective study aimed to evaluate the prognostic value of stromal content and reactive stroma in patients with breast cancer. Patients were selected from the pathology archives of Fundación Arturo López Pérez (FALP), Instituto Nacional del Cáncer (INC), and UC-CHRISTUS. Inclusion criteria were histologically confirmed invasive breast carcinoma, availability of formalin-fixed paraffin-embedded (FFPE) tissue, and available follow-up information for survival analyses; metastasis data were available for a subset of patients. Patients who received neoadjuvant therapy prior to biopsy or surgery were excluded. A total of 182 eligible patients were included. Follow-up time was defined from the date of diagnosis to the date of last follow-up or death. Clinical data collected included age, tumor size (pT), lymph node status (pN), molecular subtype (ER, PR, HER2), and histological grade.

For each case, one representative tumor block was selected by a certified pathologist. Sections (4 µm) were stained with hematoxylin and eosin (H&E) and Masson’s trichrome. In total, 182 H&E-stained and 146 Masson-stained slides were obtained and scanned using the Aperio Digital Pathology System (Aperio Technologies Inc., Vista, CA, USA) at a spatial resolution of 0.47 μm per pixel and a magnification of ×20.

### 4.2. The Total and Reactive Stroma Were Quantified Using Open-Source QuPath Software

Digital image analysis was performed using QuPath (v.0.5.0) [65]. The analysis workflow included the following steps: (1) import of .scn images and assignment of image type as brightfield H&E or brightfield (other) for Masson’s trichrome–stained sections; (2) manual annotation of tissue compartments, including tumor, stroma or reactive stroma, immune infiltrate, red blood cells, necrosis and background; (3) training of a Random Trees (RTrees) pixel classifier to automate tissue segmentation; and (4) quantification of stromal areas as follows: total stroma was calculated as the percentage of stromal area identified in H&E-stained slides relative to the total tissue area. Reactive stroma was quantified as the area corresponding to collagen deposition in Masson’s trichrome staining slides, expressed as a percentage of the stromal area previously defined on the corresponding H&E slide.

To validate reproducibility, 31 randomly selected cases were independently reviewed by two experienced pathologists. Of these, 17 cases had evaluable H&E material for total stroma validation, and 14 cases had paired evaluable Masson’s trichrome material for reactive stroma validation. The percentages of total and reactive stroma were manually estimated and compared with the QuPath-based quantification. Agreement between manual and automated measurements was assessed using Pearson’s correlation coefficient (r). All statistical analyses were performed in R (v4.4.0).

### 4.3. Stromal Stratification and Kaplan-Meier Survival Analysis

Optimal cutoff thresholds for total and reactive stromal percentages were identified via the maximally selected rank statistics method implemented in the Maxstat R package (v0.7–25). Cutoff points were determined to maximize survival differences between groups. Kaplan-Meier curves were generated via the survival (v3.7–0) and ggplot2 (v3.5.1) packages, with *p* values adjusted via the condMC method.

### 4.4. RNA Sequencing, Gene Expression Analysis and Stromal Association

Total RNA was extracted from ten 10 µm-thick FFPE sections per sample via the PureLink™ FFPE Total RNA Isolation Kit (Invitrogen, Carlsbad, CA, USA), followed by DNase I treatment to eliminate genomic DNA contamination. The RNA integrity and concentration were assessed via an Agilent TapeStation (Agilent Technologies, Santa Clara, CA, USA) and an Epoch Microplate Spectrophotometer (BioTek Instruments, Winooski, VT, USA). RNA sequencing was performed on the Illumina NovaSeq 6000 platform (Illumina Inc., San Diego, CA, USA) with paired-end 150 bp reads, targeting an average depth of ~30 million reads per sample.

The sequencing reads were aligned to the GRCh38 human reference genome via STAR aligner (v2.7.11b) [66], and gene-level expression was quantified via FeatureCounts (V2.1.0) [67]. Gene expression values were normalized, and differential expression was subsequently assessed with DESeq2 (V1.46.0) [68]. Genes showing an adjusted *p* value ≤ 0.05 and an absolute log_2_(fold change) > 1 were considered differentially expressed genes (DEGs). Downstream analyses were performed as follows: heatmaps and volcano plots for DEGs were generated with the pheatmap (v1.0.12) and EnhancedVolcano (v1.22.0) packages, whereas gene set enrichment analysis (GSEA) was computed through clusterProfiler (v4.14.6) and visualized via gseaplot2 from the enrichplot package (v1.26.6) [69,70,71]. The tumor microenvironment cell populations were quantified with an MCP-counter (v 1.2.0) [72], which was applied to the DESeq2 variance-stabilized expression matrix and specified Ensembl gene identifiers as the feature type. To investigate the relationship between gene expression and stromal content, expression values were compared between the Stroma-High and Stroma-Low groups via Student’s *t*-test (ggpubr). Pearson’s correlation analysis (cor.test, dplyr) was used to assess associations between gene expression and the percentage of the stromal area. Finally, KEGG and Reactome pathway enrichment analyses of the significant DEGs were conducted via the clusterProfiler and ReactomePA (v1.50.0) [70,73] packages.

### 4.5. Statistical Analysis

Survival analyses for overall survival (OS) and metastasis-free survival (MFS) were performed via Kaplan-Meier estimators, and survival differences between groups were evaluated via the log-rank test. Univariate and multivariate Cox proportional hazards regression models were used to estimate hazard ratios (HRs) and 95% confidence intervals (CIs) for the effects of stromal variables and clinical covariates on survival outcomes. The multivariate models included the following covariates: age, tumor size (pT), lymph node status (pN), molecular subtype (ER, PR, HER2) and histological grade. Cases with missing clinicopathological information were treated as missing values and were excluded only from analyses requiring those variables, while remaining included in all other analyses. Comparisons of clinicopathological characteristics between stromal groups were conducted via Pearson’s χ^2^ test or Fisher’s exact test, as appropriate.

To analyze gene expression differences between stromal groups, gene-wise comparisons were conducted using Student’s *t*-test or the Wilcoxon rank-sum test with the Shapiro-Wilk normality test. Multiple testing correction was applied via the Benjamini-Hochberg method (false discovery rate, FDR). Violin and box plots were generated to visualize expression distributions across groups, and adjusted *p*-values were annotated accordingly. For the selected genes, the correlation between gene expression and stromal percentage was assessed separately within the Stroma-High and Stroma-Low groups via Pearson’s correlation test. Scatter plots with linear regression lines and annotated correlation coefficients (r) and *p*-values were generated for each condition.

All statistical analyses and visualizations were performed via R (v4.4.0) with packages including ggplot2, dplyr, tidyr, ggsignif, ggpubr, and GraphPad Prism (v10.0.2, GraphPad Software, San Diego, CA, USA).

## 5. Conclusions

This study advances our understanding of tumor–stroma interactions in breast cancer by establishing the reactive stroma as a clinically relevant prognostic marker of metastasis, independent of molecular subtype. By integrating pathology and transcriptomics from FFPE tissue, we propose a clinically feasible strategy to assess stromal activity, complementing existing molecular classifiers. Future research should aim to validate these findings prospectively and explore therapeutic opportunities targeting the reactive stromal compartment.

## Figures and Tables

**Table 1:** Clinicopathological characteristics of the breast cancer cohort (*n* = 182).

Characteristic	*n* (%)
**Age**	
<55	97 (53.3%)
55–65	52 (28.6%)
>65	31 (17.0%)
Unknown	2 (1.1%)
**Menopausal status**	
Premenopausal	21 (11.5%)
Postmenopausal	44 (24.2%)
Unknown	117 (64.3%)
**ER status**	
Positive	129 (70.9%)
Negative	52 (28.6%)
Unknown	1 (0.5%)
**PR status**	
Positive	112 (61.5%)
Negative	65 (35.7%)
Unknown	5 (2.7%)
**HER2 status**	
Positive	49 (26.9%)
Negative	98 (53.8%)
Unknown	35 (19.2%)
**Histological grade**	
Well differentiated	19 (19.4%)
Moderately differentiated	51 (28.0%)
Poorly differentiated	50 (27.5%)
Unknown	62 (34.1%)
**Tumor size (pT)**	
pT0	12 (6.6%)
pT1	58 (31.9%)
pT2	26 (14.3%)
pT3	6 (3.3%)
pT4	3 (1.6%)
Unknown	77 (42.3%)
**Nodal Status (pN)**	
Positive	32 (17.6%)
Negative	71 (39.0%)
Unknown	79 (43.3%)

**Table 2:** Multivariate Cox regression analysis of reactive stromal content for overall survival and metastasis-free survival in breast cancer patients.

Variables		Overall Survival		Metastasis-Free Survival	
		HR (95% CI)	*p* Value	HR (95% CI)	*p* Value
**Age**					
	<55 years	Reference value		Reference value	
	55–65 years	2.6 (1.18–5.72)	0.018	1.22 (0.6–2.49)	0.577
	>65 years	2.87 (1.07–7.75)	0.037	4.61 (1.45–14.69)	0.010
**ER Status**					
	Positive	Reference value		Reference value	
	Negative	2.55 (0.74–8.83)	0.140	1.46 (0.39–5.41)	0.575
**PR Status**					
	Positive	Reference value		Reference value	
	Negative	1.14 (0.33–3.98)	0.838	1.27 (0.34–4.71)	0.721
**Reactive Stroma**					
	High	2.44 (0.93–6.4)	0.069	3.76 (1.91–7.39)	<0.001
	Low	Reference value		Reference value	

## Data Availability

The RNA-seq data generated in this study have been deposited in the NCBI BioProject database under accession number PRJNA1415241.

## Supplementary Materials

The following supporting information can be downloaded at: https://www.mdpi.com/article/10.3390/ijms27052213/s1.
