# Supplementary material for: Reactive Stroma as a Transversal Prognostic Biomarker for Metastasis in Breast Cancer: Integration of Digital Histopathology and Transcriptomic Profiling
Source: Int J Mol Sci. 2026 Feb 26;27(5):2213. doi: 10.3390/ijms27052213 (PMC12984561; doi:10.3390/ijms27052213)

Supplementary Table S1. Univariate Cox proportional hazards regression analysis for overall survival (OS) and metastasis-free survival (MFS) based on clinicopathological variables and total stromal content in breast cancer patients. Hazard ratios (HR), 95% confidence intervals (CI), and p-values are reported.

		**Overall Survival**		**Metastasis-Free Survival**	
**Variables**		**HR (95% CI)**	***p-value***	**HR (95% CI)**	***p-value***
**Age**					
	<55 years	Reference value		Reference value	
	55-65 years	1.75 (0.92-3.31)	0.087	1.68 (0.89-3.19)	0.110
	>65 years	4.61 (2.43-8.74)	<0.001	3.93 (1.75-8.86)	0.001
**Menopausal Status**					
	Premenopausal	Reference value		Reference value	
	Postmenopausal	0.94 (0.35-2.54)	0.902	0.78 (0.19-3.26)	0.730
**ER Status**					
	Positive	Reference value		Reference value	
	Negative	2.78 (1.64-4.71)	<0.001	2.54 (1.42-4.52)	0.002
**PR Status**					
	Positive	Reference value		Reference value	
	Negative	2.11 (1.23-3.61)	0.007	2.51 (1.39-4.54)	0.002
**HER2 Status**					
	Positive	1.41 (0.8-2.5)	0.231	2.25 (1.23-4.09)	0.008
	Negative	Reference value		Reference value	
**Histological grade**					
	Well differentiated	Reference value		Reference value	
	Moderately Differentiated	1.79 (0.51-6.36)	0.366	1.45 (0.52-4.07)	0.480
	Poorly differentiated	3.5 (1.04-11.74)	0.043	2.19 (0.8-5.98)	0.126
**Tumor size**					

	pT0	Reference value		Reference value	
	pT1	0.11 (0.02-0.63)	0.014	0.15 (0.04-0.52)	0.003
	pT2	0.58 (0.14-2.45)	0.461	0.43 (0.13-1.41)	0.163
	pT3	0.57 (0.06-5.5)	0.625	0.34 (0.04-2.93)	0.327
	pT4	7.69 (1.47-40.35)	0.016		
**Nodal Status**					
	Positive	2.28 (0.74-7.09)	0.153	1.67 (0.64-4.4)	0.297
	Negative	Reference value		Reference value	
**Total Stroma**					
	High	Reference value		Reference value	
	Low	3.49 (1.84-6.62)	<0.001	4.11 (1.62-10.4)	<0.001

Supplementary Table S2. Multivariate Cox regression analysis for overall survival (OS) and metastasis-free survival (MFS) based on total stromal content. Models were adjusted for age, pT, pN, molecular subtype, and histological grade. Hazard ratios (HR) and 95% confidence intervals (CI) are presented. Reference values are indicated where applicable.

Variables		Overall Survival		Metastasis-Free Survival	
		**HR (95% CI)**	**P-value**	**HR (95% CI)**	**P-value**
**Age**					
	<55 years	Reference value		Reference value	
	55-65 years	1.46 (0.39-5.42)	0.576	0.88 (0.3-2.6)	0.815
	>65 years	2.2 (0.37-13.05)	0.385	1.59 (0.3-8.26)	0.584
**ER Status**					
	Positive	Reference value		Reference value	
	Negative	3.07 (0.32-28.96)	0.328	1.57 (0.29-8.49)	0.598
**PR Status**					
	Positive	Reference value		Reference value	
	Negative	1.17 (0.12-11.56)	0.895	1.48 (0.29-7.47)	0.637
**Tumor size**					
	pT0	Reference value		Reference value	
	pT1	0.25 (0.04-1.78)	0.167	0.24 (0.06-0.98)	0.047
	pT2	1.07 (0.21-5.41)	0.938	0.64 (0.17-2.46)	0.517
	pT3	1.25 (0.12-12.76)	0.850	0.51 (0.05-4.96)	0.558
	pT4	5.52 (0.76-40.27)	0.092		
**Total Stroma**					
	High	Reference value		Reference value	
	Low	2.49 (0.6-10.35)	0.209	4.08 (0.88-18.94)	0.073

Supplementary Table S3. Univariate Cox proportional hazards regression analysis for overall survival (OS) and metastasis-free survival (MFS) based on clinicopathological variables and reactive stromal content in breast cancer patients. Hazard ratios (HR), 95% confidence intervals (CI), and p-values are reported.

		**Overall Survival**		**Metastasis-Free Survival**	
**Variables**		**HR (95% CI)**	**p-value**	**HR (95% CI)**	**p-value**
**Age**					
	<55 years	Reference value		Reference value	
	55-65 years	2.30 (1.06-4.98)	0.034	1.23 (0.61-2.45)	0.565
	>65 years	3.84 (1.53-9.64)	0.004	3.17 (1.26-7.96)	0.014
**Menopausal Status**					
	Premenopausal	Reference value		Reference value	
	Postmenopausal	1.12 (0.29-4.34)	0.868	0.35 (0.08-1.59)	0.174
**ER Status**					
	Positive	Reference value		Reference value	
	Negative	2.87 (1.44-5.72)	0.003	1.82 (0.97-3.43)	0.061
**PR Status**					
	Positive	Reference value		Reference value	
	Negative	2.65 (1.31-5.39)	0.007	2.42 (1.28-4.57)	0.006
**HER2 Status**					
	Positive	1.75 (0.85-3.61)	0.131	2.52 (1.3-4.87)	0.006
	Negative	Reference value		Reference value	
**Histological grade**					
	Well differentiated	Reference value		Reference value	
	Moderately Differentiated	1.27 (0.33-4.92)	0.728	1.46 (0.5-4.28)	0.489
	Poorly differentiated	2.34 (0.65-8.4)	0.192	2.31 (0.82-6.49)	0.113
**Tumor size**					

	pT0	Reference value		Reference value	
	pT1	0.11 (0.02-0.65)	0.015	0.16 (0.05-0.56)	0.004
	pT2	0.53 (0.12-2.38)	0.405	0.47 (0.13-1.77)	0.267
	pT3	0.57 (0.06-5.49)	0.624	0.41 (0.05-3.56)	0.422
	pT4	8.44 (1.33-53.79)	0.024		
**Nodal Status**					
	Positive	2.66 (0.81-8.73)	0.106	1.38 (0.49-3.88)	0.541
	Negative	Reference value		Reference value	
**Reactive Stroma**					
	High	2.51 (0.97-6.49)	0.058	3.75 (1.98-7.09)	<0.01
	Low	Reference value		Reference value	

Supplementary Figure S1. Suppression of adaptive immune processes in tumors with high reactive stromal content. (A–D) Gene Set Enrichment Analysis (GSEA) plots showing significant negative enrichment of adaptive immune processes in tumors with high reactive stromal content. Negative enrichment was observed for T cell receptor signaling pathway (A; NES = –1.85), positive regulation of T cell activation (B; NES = –2.02), positive regulation of leukocyte cell-cell adhesion (C; NES = –2.08), and positive regulation of T cell proliferation (D; NES = –1.95), suggesting a potential suppression of T cell–mediated immune responses in this patient group.

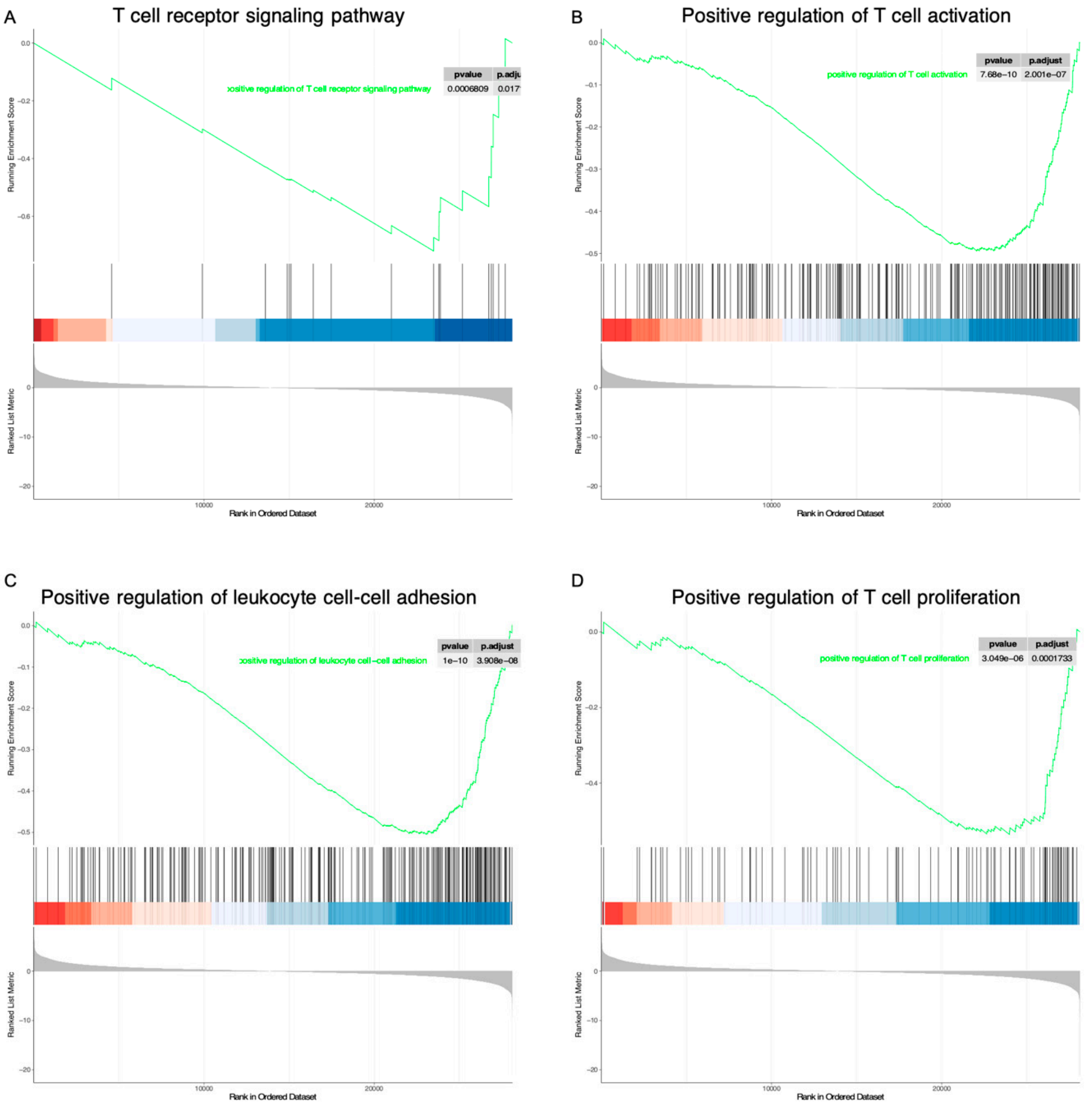

Supplement: Supplementary file 1 [file ijms-27-02213-s001.zip › ijms-4089805-supplementary.pdf]
